# Hand hygiene practices among Jordanian nurses in Amman

**DOI:** 10.4314/ahs.v22i3.76

**Published:** 2022-09

**Authors:** Ahmad M Saleh, Saud M Alrawaili, Walid Kamal Abdelbasset

**Affiliations:** 1 Department of Nursing, College of Applied Medical Sciences, Prince Sattam Bin Abdulaziz University, Al-Kharj, Saudi Arabia; 2 Department of Health and Rehabilitation Sciences, College of Applied Medical Sciences, Prince Sattam Bin Abdulaziz University, Al-Kharj, Saudi Arabia; 3 Department of Physical Therapy, Kasr Al-Aini Hospital, Cairo University, Giza, Egypt

**Keywords:** Hand hygiene, Health care professionals, Alcohol-based hand rub, Hand washing

## Abstract

**Background:**

Hand hygiene is a simple and effective practice that helps to reduce the spread of hospital-acquired infections. However, health care professionals' adherence to hand hygiene guidelines is low. The purpose of this study is to evaluate hand hygiene practices among Jordanian nurses working in hospitals.

**Methodology:**

The standardized version of the World Health Organization (WHO) questionnaire was given to Jordanian nurses from two hospitals in Amman.

**Result:**

The response rate was 76 percent, with 173 nurses contacted to enroll 226 participants. According to the study, 65.5 percent (113) of the participants have a good practice hand hygiene, while 11 percent (19) practiced poor hand hygiene. The percentage of female participants who practiced good hand hygiene was found to be significantly higher (70 percent) than the percentage of male participants (30 percent).

**Conclusion:**

To improve compliance with hand hygiene practices, male nurses and nurses working in the department of internal medicine and pediatrics need in-service educational intervention. Posters and other visual aids emphasizing the importance of hand hygiene should be displayed in all departments to raise awareness of the importance of hand hygiene among nurses.

## Introduction

Hand hygiene is a simple but critical method for preventing the spread of healthcare-associated infections in hospital settings. Hand hygiene is a cost-effective intervention that plays an important role in infection control and patient safety in health care settings.^1^ It is estimated that hospital acquired infections affect more than 1.4 million people worldwide.^2^.

According to the World Health Organization, the prevalence of hospital acquired infections is approximately 5–10% in developed countries and approximately 40% in developing countries.^3^ According to previous research, the burden of hospital acquired infection is disproportionately high in developing countries.^4^ Previous research has shown that washing hands with soap and water or using an alcohol-based hand rub are the most effective methods for preventing the spread of infectious diseases.^5^

There is ample evidence from previously published literature indicating that infections can have serious negative effects on patients, resulting in prolonged hospital stays, pneumonia, and even death.^6^ Furthermore, the presence of hospital acquired infections increases the economic burden on families as well as the cost of the country's health care delivery system.^7^ Health care providers' hands are often infected with pathogenic microbial species, which play an important role in the transmission of infections from one person to another within the hospital setting.^8^

Nurses make up the majority of health care providers and are regarded as the nucleus of the health care delivery system because they have direct contact with patients and spend more time with them.^9^–^11^ Previous research has shown that strict hand hygiene practices among health care providers will greatly decrease the incidence of hospital acquired infections.^12^–^15^ Several studies, however, show that hand hygiene compliance among health care providers is commonly low.^16^, ^17^ In addition, a study conducted in Malawian hospitals by Kalata et al.^18^ discovered that only 23% of health care providers practiced hand hygiene.

Hand hygiene adherence is a significant impediment to developing infection control programs in hospitals. Furthermore, no perfect techniques for tracking compliance with hand hygiene practices among health care providers are available.^19^ As a result, the aim of this research is to determine hand hygiene practices among nurses to discover the relationship between hand hygiene practices and general demographic characteristics of nurses.

## Materials And Methods

### Ethical Approval

Ethical approval was obtained from the research ethics committee of both hospitals. All participants participated on a voluntary basis and gave their informed consent.

### Study Setting & Participants

From May 2018 to August 2018, a cross-sectional survey was administered to nurses in two chosen hospitals in Jordan's Amman area. To find hospitals for the study, a convenience sampling was used. One governmental and one private hospital were included in the study because their managers were available and facilitated the data collection process. The research participants were recruited using an opportunistic sampling methodology. Nurses who had direct contact with patients at the chosen hospitals were asked to participate in the study.

### Sample Size Calculation

The Raosoft sample size calculator was used to determine the sample size. The margin of error was held at 5%, with a confidence interval of 95%. The computed minimum sample size was 146.

### Data Collection

During their break time, the study principal investigator visited the chosen hospitals and had face-to-face discussions with nurses specific to particular departments of the hospital. The nurses were briefed on the studies and reminded that their participation was entirely voluntary. The principal investigator also informed the nurses that the information gathered would be used solely for research purposes and would be kept strictly secret. At the time of enrollment, each nurse provided written informed consent.

Data was compiled using standard questions about hand hygiene practice based on the WHO Guidelines on Hand Hygiene for Health Care Professionals.^20^,^21^ Additional questions were added to obtain general demographic information from participants, such as age, gender, department, and years of experience.

The questionnaire was created in English and then translated into Arabic for the purposes of study. All of the nurses from the chosen hospitals were given an online link where they could access the electronic version of the questionnaire in Arabic. The nurses were asked to complete the questionnaire online and submit it.

### Development of Questionnaire

The questionnaire contained 11 questions, each with a score of 1 or 0 for correct or incorrect answers. To lessen the impact of social desirability, if a participant answered "sometimes,” it was considered a wrong answer. The cumulative score varied between 0 and 11. The total score was split into three categories: ‘inadequate practice’ (less than 6), ‘adequate practice’ (6 to 8) and ‘good practice' (more than 8 up to 11).

- Good Practice: More than 75% of study participants answered to the practice questions in accordance with the recommended hand washing practice.

- Adequate Practice: Only 51% -74% of study participants responded to the practice questions in accordance with the recommended hand washing practice.

- Poor Practice: Only 50% of study participants responded to the practice questions in accordance with the recommended hand washing practice.

### Data Analysis

SPSS was used to do the data analysis (Version 21.0, SPSS Inc. Chicago, IL, USA). A frequency table was used to identify nurses' self-reported hand hygiene practices. As necessary, the Chi-square test was applied to nominal scales. The p-value of 0.05 was deemed statistically important.

## Results

Two hundred and twenty-six nurses from the two hospitals were contacted to hire 173 nurses for the study, yielding a 76 percent response rate. The general demographic features of the study participants are shown in **Table 1**: Among the 173 participants, 51 percent (88) were between the ages of 20 and 29, and 30 percent (52) had clinical experience ranging from 1 to 9 years. The majority of the attendees, 80 percent (138), had gone through a hand was hing training course.

**Table 1:** General demographic characteristics of nurses who responded practice questionnaire regarding hand hygiene (N=173)

Variable	(%)
Gender Male Female	52 (30%) 121 (70%)
Age (years) 20–29 30–39 >40	88 (51%) 52 (30%) 92 (81%)
Department Internal medicine Surgery Intensive care unit Mixed medical/surgical Emergency unit Pediatrics	23 (13.3%) 31 (17.9%) 22 (12.7%) 42 (24.3%) 32 (18.5%) 23(13.3%)
Working Experience (years) 1–9 10–19 >20	52 (30%) 72 (42%) 49 (28%)
Attend a training course about hand washing Yes No Not Sure	138 (80%) 22 (12.8%) 13 (7.2%)

**Table 2:** Summarizes the responses of participants to the practice questionnaire. Almost 77.5 percent (134) of the participants said they wash their hands on a regular basis, and 70.5 percent (122) said they use alcohol-based hand rubs.

**Table 2:** Nurses responses to the practice questionnaire (N=173)

Hand Hygiene Practices	(%)
Do you routinely wash your hands?	
Yes	134 (77.5%)
No	13 (7.5%)
Sometimes	26 (15%)
Do you routinely use an alcohol-based hand rubs for hand hygiene?	
Yes	122 (70.5%)
No	22 (12.8%)
Sometimes	29 (16.8%)
Do you wash your hands in the following situation?	
After physical contact patient with patients	
Yes	122 (70.5%)
No	19 (10.9%)
Sometimes	32 (18.5%)
After exposure to body fluids	
Yes	109 (63%)
No	12 (6.9%)
Sometimes	52 (30%)
Before any medical procedures	
Yes	163 (94.2%)
No	10 (5.8%)
Sometimes	0 (0%)
If they look or feel dirty	
Yes	156(90%)
No	9 (5.2%)
Sometimes	8 (4.6%)
Before entering an isolation roomYes	
No	140 (80.9%)
Sometimes	15 (8.7%)
After exiting an isolation room	18 (10.4%)
Yes	
No	135 (78%)
Sometimes	21 (12.14%)
After removing gloves	17 (9.8%)
Yes	
No	150 (86.7%)
Sometimes	11 (6.36%)
After going to the toilet	12 (6.93%)
Yes	
No	145 (83.8%)
Sometimes	14 (8.1%)
After touching potentially contaminated objects	14 (8.1%)
Yes	
No	133 (76.88%)
Sometimes	7 (4.05%)
	33 (19.1%)

The highest percentages of hand washing practice reported by participants were 94.2 percent (163) before any medical procedures, 90 percent (156) when their hands appeared dirty, and 83.8 percent (145) after going to the toilet. Participants indicated the lowest percentages of hand washing practice reported by 63 percent (109) after exposure to body fluids, 76.88 percent (133) after touching potentially contaminated objects, and 78 percent (135) after existing an isolation room.

**Table 3:** Depicts the distribution of participants based on their hand hygiene performance. According to the study's self-reported responses to the hand hygiene questionnaire, 65.5 percent (113) of the participants practiced good hand hygiene (>75 percent responses are in line with the suggested hand washing practice), while 11 percent (19) practiced in adequate hand hygiene (Only 50% of study participants responded to the practice questions in accordance with the recommended hand washing practice).

**Table 3:** Distribution of nurses according to performance of hand hygiene (N=173).

Hand Hygiene Practice Score	(%)
Inadequate Practice (<6)	19 (11%)
Adequate Practice (6–8)	41 (23.5%)
Good Practice (9–11)	113 (65.5%)

**Table 4:** Shows the relationship between participants' self-reported hand hygiene practices and general demographic factors. The study showed that good hand hygiene practice was substantially higher among female participants (62.8 percent) than male participants (36.5 percent). Participants aged 20–29 years had a considerably lower proportion of good hand hygiene practice (42.6.1 percent) than those aged more than 40 years (50 percent). Participants with more than 15 years of experience had a substantially lower proportion of good hand wash practice (36.9 percent) than those with 1–5 years of experience (39.3 percent). Participants who practiced excellent hand hygiene (86.7 percent) had considerably more exposure to the training course than participants who practiced proper hand hygiene (13.3 percent).

**Table 4:** Correlation between the practice of nurses regarding hand hygiene and general demographic variables (N=173)

Variables	Hand Hygiene Practices Score			Total N (Percent)
	Inadequate Practice (1)	Adequate Practice (2)	Good Practice (3)	
Gender Male Female	14 (30.8%) 32 (26.4%)	17 (32.7%) 13 (10.8%)	19 (36.5%) 76 (62.8%)*	52 (30%) 121 (70%)
Age 20–29 30–39 > 40	9 (16.6%) 9 (15.3%) 10 (16.7%)	22 (40.8%) 24 (40.7%) 20 (33.3%)	23 (42.6%) 26 (44.1%) 30 (50%)	54 (31%) 59 (34%) 60 (35%)
Years of Experience 1–5 Years 6–10 Years 11–15 Years >15 Years	13 (23.2%) 14 (18.4%) 7 (31.8%) 6 (31.5%)	21 (37.5%) 17 (22.4%) 5 (22.7%) 6 (31.5%)	22 (39.3%) 45 (59%) 10 (45.5%) 7 (36.9%)*	56 (32.1%) 76 (43.4%) 22 (12.7%) 19 (10.9%)
Attend a training course about hand washing Yes No	32 (21.3%) 5 (15.7%)	40 (26.7%) * 8 (21.9%)	78 (52%) * 20 (62.5%)	150 (87%) 33 (13%)

* P value is significant at the level < 0.05

# Difference in number of total participants due to removal of participants who were not sure about attending training course for hand washing

## Discussion

The current research was designed to look into the self-reported hand hygiene practices of nurses in Amman. The World Health Organization (WHO) has developed a questionnaire to improve hand hygiene compliance and to decrease the incidence of hospital-acquired infections.^22^

The main findings of the current study found that 65.5 percent of nurses have practiced a good hand hygiene and 23.5 percent practiced adequate hand hygiene. However, the current study's rates of insufficient or poor hand hygiene practice 11 percent. The differences in hand hygiene practice may be attributed to differences in the subjects enrolled in the experiments. Kendall et al.^23^ found in a review article that hand hygiene compliance among health care workers is low.^23^ Similarly, two other studies 24,25 found that nurse hand hygiene compliance is low and heavily affected by a heavy workload, a high number of clinical procedures, and skin conditions. Furthermore, some studies conducted in Jordan, Sri Lanka, Ethiopia, and other nations revealed that the rate of hand hygiene compliance among health care workers ranges from 5.53 percent to 87.5 percent.^20^, ^26^–^31^

In terms of the relationship between gender and self-reported hand hygiene practices, the current study found that female nurses had substantially greater good hand hygiene practices than male nurses. These findings are consistent with previous research, which found similar substantial differences in hand hygiene performance between males and females.^32^–^35^ Similarly, Cruz et al. discovered that the mean score for hand hygiene practice differs between males and females.^36^ However, females' higher compliance rate with hand hygiene may be related to their proclivity to engage in socially acceptable behaviors.^37^, ^38^ In the current study, 87 percent of nurses self-reported having completed hand hygiene training courses, which was mirrored in a higher proportion of good hand wash practice (62.8 percent). According to a study conducted by Mazi et al., 39 on 1,975 health care workers, there was an increase in hand hygiene compliance from 51 percent to 67 percent following an intervention campaign from May to June 2010. According to Cruz et al., there is a need for gender-specific educational intervention to address gender disparity.^36^

Randle et al.,^40^ affirmed that regular hand hygiene training using various instructional materials to remind health care workers of hand hygiene would greatly improve hand hygiene practice among health care workers. Shinde et al.,^41^ called for frequent hand hygiene training programs using World Health Organization materials, as well as active involvement of infection control teams in order to raise awareness about hand hygiene and establish a supportive environment for improving hand hygiene practice in hospitals.

The current study has some strengths including its consideration for tracking compliance with hand hygiene practices among health care providers are available and high response rate (76%). On other hand, some limitations have been observed in the study such as the findings are based on self-reported questionnaires. Hand hygiene compliance among nurses, particularly those who have completed the hand washing training course, may be exaggerated. Furthermore, since the research was carried in a hospital setting among nursing practitioners, there is a chance of social desirability bias.^42^

As a result, the participants' response “sometimes” was interpreted as a negative response in order to mitigate the impact of social desirability. Another limitation of the study is that no proxy measures, such as hospital-acquired infection rates in different departments or nurses' access to hand hygiene facilities in different departments, were assessed in order to verify self-reported hand hygiene practice. However, according to a previous study,^43^ the self-reported hand hygiene compliance rate can be considered an appropriate replacement where cost and time restrictions exist. Finally, the study's participants were recruited using the opportunistic sampling methodology, which limits the generalizability of the results.

Hands will be one of the main ways that diseases can be spread 80% of communicable diseases. Therefore, Awareness about proper hand hygiene should be the most accessible and necessary tools to stop widespread loss of life.

## Conclusion

The purpose of this study was to determine nurses' self-reported hand hygiene practices. In this study, 62.8 percent of nurses practiced good hand hygiene. Male nurses and nurses working in the department of internal medicine need in-service hand hygiene education to improve their compliance with hand hygiene practices. Posters and other visual aids emphasizing the importance of hand hygiene should be displayed in all departments to raise awareness of the importance of hand hygiene among nurses.

## References

[1] Colet PC, Cruz JP, Cruz CP, Al-Otaibi J, Qubeilat H, Alquwez N (2015). Patient safety competence of nursing students in Saudi Arabia: A self-reported survey. Int J Health Sci (Qassim).

[2] Sallami ZA (2016). Assessment of hand hygiene attitude, knowledge and practice among health science students in aden university. J Biosci and Med.

[3] World Health Organization Improved hand hygiene to prevent health care associated infections.

[4] Al Kadi A, Salati SA (2012). Hand hygiene practices among medical students. Interdiscip Perspect Infect Dis.

[5] Anderson JL, Warren CA, Perez E (2008). Gender and ethnic differences in hand hygiene practices among college students. Am J Infect Control.

[6] Glance LG, Stone PW, Mukamel DB, Dick AW (2011). Increases in mortality, length of stay, and cost associated with hospital-acquired infections in trauma patients. Arch Surg.

[7] Zimlichman E, Henderson D, Tamir O (2013). Health care-associated infections: a meta-analysis of costs and financial impact on the US health care system. JAMA Intern Med.

[8] Gilbert K, Stafford C, Crosby K, Fleming E, Gaynes R (2010). Does hand hygiene compliance among health care workers change when patients are in contact precaution rooms in ICUs?. Am J Infect Control.

[9] Buerhaus PI, Auerbach DI, Staiger DO (2007). Recent trends in the registered nurse labor market in the U.S.: Short-run swings on top of long-term trends. Nurs Econ.

[10] Abualrub RF (2007). Nursing shortage in Jordan: what is the solution?. J Prof Nurs.

[11] Elhajji FD, Al-Taani GM, Anani L, Al-Masri S, Abdalaziz H, Su'ad H, Al Bawab AQ, Scott M, Farren D, Gilmore F, Versporten A (2018). Comparative point prevalence survey of antimicrobial consumption between a hospital in Northern Ireland and a hospital in Jordan. BMC health services research.

[12] Sabra SM, Abdel-Fattah MM (2012). Epidemiological and microbio-logical profile of nosocomial infection in Taif hospitals, KSA (2010-2011). World J Med Sci.

[13] Al-Tawfiq JA, Abed MS, Al-Yami N, Birrer RB (2013). Promoting and sustaining a hospital-wide, multifaceted hand hygiene program resulted in significant reduction in health care-associated infections. Am J Infect Control.

[14] Mestre G, Berbel C, Tortajada P (2012). “The 3/3 strategy”: a successful multifaceted hospital wide hand hygiene intervention based on WHO and continuous quality improvement methodology. PLoS One.

[15] Rosenthal VD, Guzman S, Safdar N (2005). Reduction in nosocomial infection with improved hand hygiene in intensive care units of a tertiary care hospital in Argentina. Am J Infect Control.

[16] Thakker VS, Jadhav PR (2015). Knowledge of hand hygiene in undergraduate medical, dental, and nursing students: A cross-sectional survey. J Family Med Prim Care.

[17] Ariyarathne MH, Gunasekara TD, Weerasekara MM, Kottahachchi J, Kudavidanage BP, Fernando SS (2013). Knowledge, attitudes and practices of hand hygiene among final year medical and nursing students at the University of Sri Jayewardenepura. Sri Lankan Journal of Infectious Diseases.

[18] Kalata NL, Kamange L, Muula AS (2013). Adherence to hand hygiene protocol by clinicians and medical students at Queen Elizabeth Central Hospital, Blantyre-Malawi. Malawi Med J.

[19] Eveillard M, Hitoto H, Raymond F (2009). Measurement and interpretation of hand hygiene compliance rates: importance of monitoring entire care episodes. J Hosp Infect.

[20] Cruz Jonas P, Bashtawi Meshrif A (2016). Predictors of hand hygiene practice among Saudi nursing students: Across-sectional self-reported study. J Infect Public Health.

[21] Hand Hygiene WHO (2009). Why, How & When? Patient safety, a world alliance for safer health care, save lives, cleans your hands A questionnaire developed by infection control programme, Hôpitaux Universitaires de Genève English Version.

[22] (2009). WHO Guidelines on Hand Hygiene in Health Care First Global Patient Safety Challenge Clean Care is Safer Care.

[23] Kendall A, Landers T, Kirk J, Young E (2012). Point-of-care hand hygiene: preventing infection behind the curtain. Am J Infect Control.

[24] Abd Elaziz KM, Bakr IM (2009). Assessment of knowledge, attitude and practice of hand washing among health care workers in Ain Shams University hospitals in Cairo. J Prev Med Hyg.

[25] Jang JH, Wu S, Kirzner D (2010). Focus group study of hand hygiene practice among healthcare workers in a teaching hospital in Toronto, Canada. Infect Control Hosp Epidemiol.

[26] Aledeilah R, Abo el-Fetoh N, Albaker A (2018). Assessment of Knowledge, Attitude and Practice of Hand Hygiene among Health Care Workers in Arar City, Saudi Arabia. Egyptian Journal of Hospital Medicine.

[27] AlOmari F, AlQarny M (2015). Knowledge and practice of hand hygiene among healthcare workers at Armed Forces Military Hospitals, Taif, Saudi Arabia. Int J Med Sci Public Health.

[28] Engdaw GT, Gebrehiwot M, Andualem Z (2019). Hand hygiene compliance and associated factors among health care providers in Central Gondar zone public primary hospitals, Northwest Ethiopia. Antimicrob Resist Infect Control.

[29] Jemal S (2018). Knowledge and Practices of Hand Washing among Health Professionals in Dubti Referral Hospital, Dubti, Afar, Northeast Ethiopia Advances in preventive medicine.

[30] Goyal L, Kumar A, Goyal T (2018). Knowledge, attitude and practices towards hand hygiene among nursing staff working in a tertiary care setting in north India: a Descriptive cross-sectional study. Eur J Pharm Med Res.

[31] Sharif A, Arbabisarjou A, Balouchi A, Ahmadidarrehsima S, Kashani HH (2016). Knowledge, Attitude, and Performance of Nurses toward Hand Hygiene in Hospitals. Glob J Health Sci.

[32] Ahmed J, Malik F, Memon ZA (2020). Compliance and Knowledge of Healthcare Workers Regarding Hand Hygiene and Use of Disinfectants: A Study Based in Karachi. Cureus.

[33] Elkhawaga G, El-masry R (2017). Knowledge, Beliefs and Self-reported Practices of Hand Hygiene among Egyptian Medical Students: Does Gender Difference Play a Role?. Journal of Public Health in Developing Countries.

[34] Arikan Akan O, Cetinkaya Y, Ozgultekin A (2004). National multi-center study to evaluate the baseline hand washing compliance in the intensive care units of three Turkish hospitals: differences between genders. Am J Infect Control.

[35] Kinnison A, Cottrell RR, King KA (2004). Proper hand-washing techniques in public restrooms: differences in gender, race, signage, and time of day. Am J Health Educ.

[36] Cruz J, Cruz C, Al-Otaibi A (2015). Gender differences in hand hygiene among Saudi nursing students. Int J Infect Control.

[37] Johnson HD, Sholcosky D, Gabello K, Ragni R, Ogonosky N (2003). Sex differences in public restroom hand-washing behavior associated with visual behavior prompts. Percept Mot Skills.

[38] Suen LKP, So ZYY, Yeung SKW, Lo KYK, Lam SC (2019). Epidemiological investigation on hand hygiene knowledge and behaviour: a cross-sectional study on gender disparity. BMC Public Health.

[39] Mazi W, Senok AC, Al-Kahldy S, Abdullah D (2013). Implementation of the world health organization hand hygiene improvement strategy in critical care units. Antimicrob Resist Infect Control.

[40] Randle J, Clarke M, Storr J (2006). Hand hygiene compliance in healthcare workers. J Hosp Infect.

[41] Shinde M, Mohite V (2014). A Study to Assess Knowledge, Attitude and Practices of Five Moments of Hand Hygiene among Nursing Staff and Students at a Tertiary Care Hospital at Karad. Int J Sci Res (Ahmedabad).

[42] Kupfer TR, Wyles KJ, Watson F, La Ragione RM, Chambers MA, Macdonald AS (2019). Determinants of hand hygiene behaviour based on the Theory of Interpersonal Behaviour. J Infect Prev.

[43] Vikke HS, Vittinghus S, Betzer M (2019). “Hand hygiene perception and self-reported hand hygiene compliance among emergency medical service providers: A Danish survey”. Scand J Trauma Resusc Emerg Med.

